# Bilayer Lipid Membrane as Memcapacitance: Capacitance–Voltage Pinched Hysteresis and Negative Insertion Conductance

**DOI:** 10.3390/membranes13010097

**Published:** 2023-01-11

**Authors:** Elena Yu. Smirnova, Andrey A. Anosov

**Affiliations:** 1 The Department of Medical and Biological Physics, Sechenov First Moscow State Medical University (Sechenov University), 119991 Moscow, Russia; 2 Kotelnikov Institute of Radioengineering and Electronics of RAS, 125009 Moscow, Russia

**Keywords:** bilayer lipid membranes, non-linear capacitance, pinched hysteresis loops, memcapacitance, negative conductance

## Abstract

Inelastic (dissipative) effects of different natures in lipid bilayer membranes can lead to hysteresis phenomena. Early, it was shown that lipid bilayer membranes, under the action of a periodic sinusoidal voltage, demonstrate pinched-hysteresis loops in the experimental capacitance–voltage dependences and are almost the only example of the physical implementation of memcapacitance. Here, we propose an equivalent circuit and mathematical framework for analyzing the dynamic nonlinear current response of a lipid bilayer membrane as an externally controlled memcapacitance. Solving a nonlinear differential equation for the equivalent circuit of a membrane in the form of a parallel connection of a nonlinear viscoelastic capacitor and an active resistance using the small parameter method, we obtain explicit analytical dependences for the current response of the membrane and pinched-hysteresis loops. The explicit solutions and their comparison with experimental data allow us to identify the lumped equivalent circuit parameters that govern the memcapacitor behavior of the membrane and hence the magnitude of the hysteresis. We quantify the memcapacitance hysteresis in terms of negative work done by the control signal. An analysis of the formulas leads to the conclusion that the determining factor for the appearance of pinched hysteresis is the type of nonlinear dependence of the device capacitance on voltage.

## 1. Introduction

The electric properties of biomembranes play a pivotal role in cellular functions. The parameters of electrical equivalent circuits of biological and model bilayer membranes—capacitance and conductance and their dependence on external influences—have been intensively studied over the past 50 years. The dependence of the capacitance of lipid bilayer membranes with various solvents on the electric field was studied in early experiments [1,2,3,4,5,6]. The increase in capacitance *C* under the action of an electric field was described by the empirical formula $C=C_{0}\left(1+\beta U^{2}\right)$. The coefficient of the nonlinearity of capacitance β for membranes with a solvent was estimated at 2–10 V^−2^, for membranes without a solvent (“dry”) membranes 0.022 V^−2^ [1]. In studies of the nonlinear capacitance of the droplet interface bilayer (DIB), the value of the coefficient of nonlinearity depended on the type of solvent and was 25.8 V^−2^ for membranes of diphytanoylphosphatidylcholine (DPhPC) in decane and 1.75 V^−2^ in hexadecane [7,8]. The value of the coefficient for cells was estimated to be 0.108–0.135 V^−2^ [9]. The change in the capacitance of membranes under the action of an electric field is associated with various processes—electrostriction, a change in the thickness of the membrane as a result of solvent exclusion, membrane area increase [1,2,3,4,5,6], and thermal fluctuations [10]. These processes have different relaxation times and, under the action of periodic electric fields, should manifest themselves in different frequency ranges. The dynamic current response of the membrane and its equivalent parameters must depend on the frequency of the alternating voltage. The nonlinearity coefficient β is expected to be maximum at low frequencies, since in this case all the processes occurring under the action of the electric field and responsible for the nonlinearity have time to take place. Inelastic (dissipative) effects of various natures, causing a delay in the polarization of the membrane and capable of causing hysteresis phenomena, should also manifest themselves in different frequency ranges.

Hysteresis effects on the current responses of the membrane to a periodic triangular voltage were noted in early experiments [5,6]. These phenomena are also discussed in works where DIB is used as a biological membrane model and dynamic capacitive currents in bilayer membranes with different solvents are studied [7,8,11]. It was shown in [11] that the existence of hysteresis is associated with voltage-dependent changes in the geometry of the bilayer, which depend on the viscosity of the solvent (decane and hexadecane were compared), temperature and the frequency of the command sinusoidal voltage. The authors first drew attention to a new aspect of studying the dynamic electrical characteristics of bilayer lipid membranes, showing that lipid DIB membranes with a solvent under the action of a periodic sinusoidal voltage exhibit the properties of memcapacitive (memory capacitive) systems. The concept of memristic systems proposed in [12] was extended to capacitive elements—capacitors, the properties of which depend on the state and history of the system. A change in the capacitance of such systems under the action of an external control voltage can be associated with geometric changes in the systems and the influence of inelastic (dissipative) effects [13]. Memcapacitors exhibit narrowed hysteresis loops in charge–voltage dependence. It is assumed that these systems can be used as synapses in artificial neural networks [14,15,16,17]. DIB membranes showing pinched hysteresis loops in charge–voltage coordinates, as well as in experimental capacitance–voltage dependences [11], are almost the only example of the physical implementation of memcapacitance.

To evaluate the parameters characterizing the dynamic electrical properties of the model and biological membranes, periodic command voltage of various types is applied to them in voltage clamp mode: triangular or sinusoidal voltage, rectangular pulses, voltage ramp stimulus, or combinations thereof [18]. Interpretation of experimental responses to these signals depends on the choice of an adequate equivalent circuit that allows one to estimate the characteristics of the membrane with varying accuracy. Traditionally, a membrane is thought of as a parallel connection between a capacitor and a resistor. Series resistance (access resistance) in the equivalent circuit of the membrane allows for a more accurate estimation of membrane parameters [19,20]. However, there are few models that describe the dynamic electrical behavior of membranes with non-linear capacitances. Basically, for the theoretical description of nonlinear and transient processes in the current response of the membrane, the capacitances of the classical equivalent circuit are assumed to be exponentially dependent on time in an explicit form [21,22] or described by first-order differential equations [10,11].

In this paper, we introduce a mathematical basis for interpreting experimental observations of the BLM input-output dynamics as memcapacitors with external control of a triangular-shaped periodic signal. For an analytical description of memcapacitor properties, we propose an equivalent circuit of a membrane in the form of a parallel connection of a viscoelastic capacitor [23] and resistance modeling of the ion permeability of membranes. The ion permeability can increase in the presence of protein or lipid channels and affect the memcapacitance properties. Compiling a nonlinear differential equation for the equivalent circuit and solving it using the small parameter method, we obtain explicit analytical dependences of the current response of the membrane and pinched-hysteresis loops on time and applied voltage.

A comparison of analytical expressions with experimental current recordings of membranes confirms the adequacy of the equivalent circuit and the mathematical model. Explicit solutions for the current–voltage characteristics and pinched-hysteresis loops in comparison with the experimental data make it possible to estimate the parameters of the equivalent circuit and their dependence on the characteristics of the input signal and the properties of the membrane. The resulting formulas allow us to determine how these parameters control the memcapacitance behavior of the membrane and the amount of hysteresis in the capacitance–voltage coordinates.

We also quantify pinched hysteresis in terms of the work done by the control voltage, for which we obtain explicit formulas in terms of equivalent circuit parameters and control signal characteristics. Interpretation of the properties of a lipid bilayer membrane in the context of memcapacitance systems allows us to define a lipid bilayer membrane as an active memcapacitive device. The nonlinearity of a capacitance of a certain type, combined with viscous dissipative processes that cause a delay in membrane polarization, leads to the appearance of an insertion negative conductance proportional to the width of the hysteresis loop.

## 2. Materials and Methods

### 2.1. Electrical Model of a Viscoelastic Membrane: Solving a Nonlinear Differential Equation

The main processes leading to a noticeable non-linear change in the capacitance of a planar bilayer lipid membrane under an electric field are deformation of the membrane and a decrease in its thickness due to solvent extrusion, as well as an increase in the area of the membrane. These processes proceed quite slowly (relaxation times of solvent extrusion are proportional to its viscosity and amount to 5 ms–5 s [5,24,25,26]. When a cyclic electric field of corresponding frequencies is applied, these processes affect the dynamic behavior of the geometric dimensions of the membrane and, accordingly, its equivalent electrical parameters.

**Figure 1:**
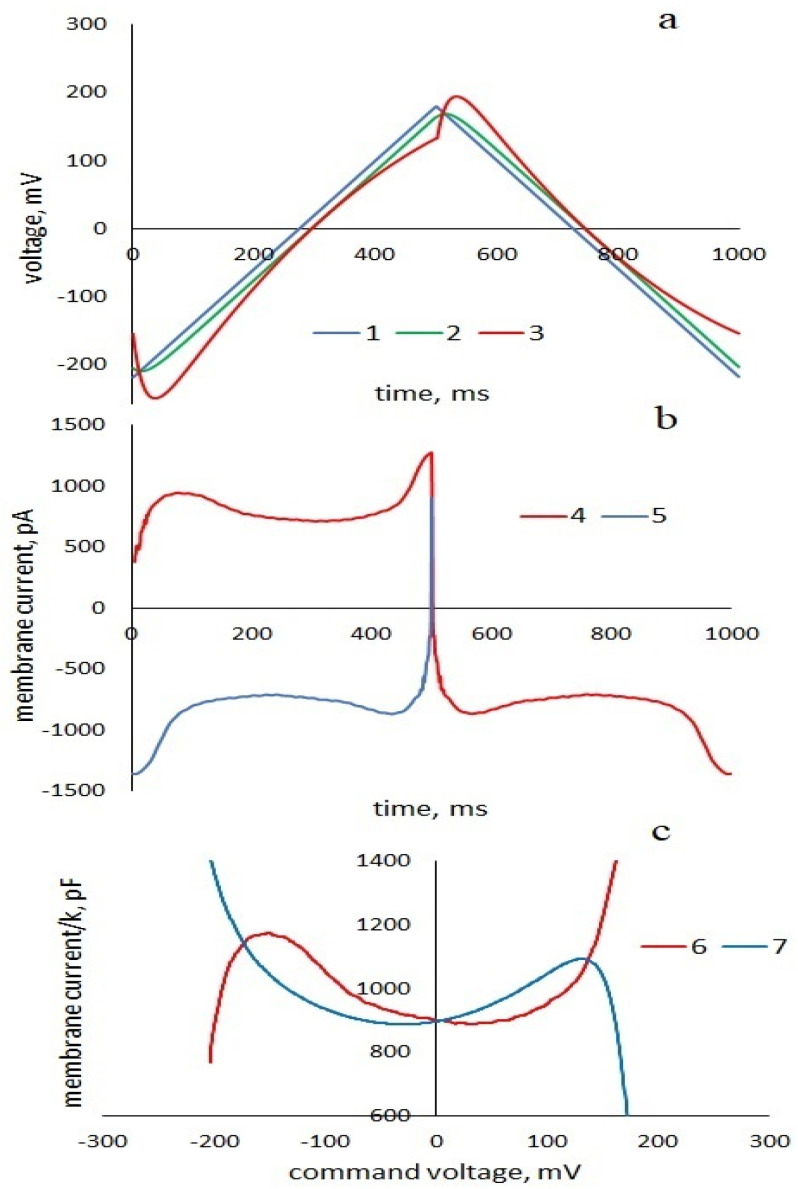
(**a**): 1—command triangular voltage with a frequency of 1 Hz and an amplitude of 200 mV; 2—voltage $\tilde{U_{0}}$**,** calculated by Formulas (S9) and (S10), proportional to the input signal; 3—nonlinear ${\tilde{U}}_{up}$ and ${\tilde{U}}_{down}$, calculated by Formulas (8) and (9). (**b**): Non-linear experimental current response of the membrane to the commanded triangular voltage, 4: $I_{0,up}\left(t\right)$, $I_{0,down}\left(t\right)$, 5: $I_{0,down,\;inv}\left(t\right)$; (**c**): experimental pinched-hysteresis loops: 6: $I_{0,up}\left(U\right)/k\;$, 7: $I_{0,down,\;inv}\left(U\right)/-k$.

**Figure 2:**
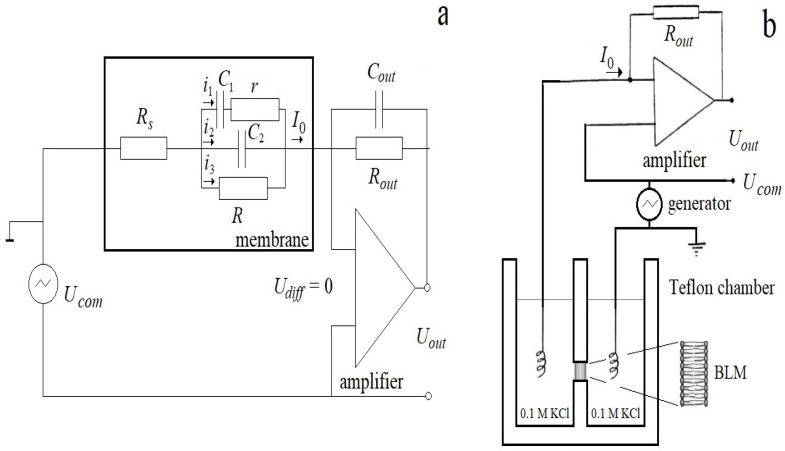
Equivalent circuit of the membrane (**a**) and measuring system (**b**).

To analytically describe the dynamic current response of the membrane (Figure 1) to a periodic triangular voltage with frequencies corresponding to the relaxation times, we consider the membrane as a viscoelastic capacitor [23]. Following the logic of this work, let us expand the total current response of the membrane into that caused by a purely elastic change in geometry and a non-linear current that depends on the prehistory of the process. The circuit of this current imitates the viscoelastic response of a section of the membrane in which the electrical displacement and polarization lag behind the field strength and depend on its value at the previous time. This occurs when the electric polarization does not have time to follow the changes in the electric field. The equivalent circuit of the membrane is shown in Figure 2. The equivalent parameters of the membrane are the series connection of resistance r, proportional to the viscosity of the solvent, and the nonlinear capacitance $C_{1}=\left(1-\varkappa \right)C_{0}\left(1+\beta {\tilde{U}}^{2}\right).$ The material parameter $\left(1-\varkappa \right)$ represents physically the fraction of the membrane, which in a given time range has time-independent deformation (solvent), β is the coefficient of capacitance nonlinearity for this part of the membrane, $C_{0}$ is the capacitance of the entire membrane at zero voltage, $\tilde{\;U}$ is the voltage across the capacitance $C_{1}$*,* physically proportional to the electrical displacement. Thus, the current circuit $i_{1}$ represents a part of the membrane; the deformation and the corresponding change in capacitance are time-dependent processes due to viscous processes that prevent changes in the membrane geometry. The corresponding equation for the current is given by:

(1) $$i_{1}=\left(1-\varkappa \right)C_{0}\left(1+\beta {\tilde{U}}^{2}\right)\frac{d\tilde{U}}{dt}\;$$

The capacitance in the second branch $C_{2}=\varkappa C_{0}\left(1+\beta \prime U^{2}\right)$ is the capacitance of the part of the membrane that gives a purely elastic response, the nonlinearity of which can be neglected, since the coefficient of nonlinearity of “dry” membranes without solvent β′≪β [1]. The membrane is purely elastic when ϰ = 1. The current in this branch is

(2) $$i_{2}=\varkappa C_{0}\frac{dU}{dt}\;$$

where *U* is the command voltage proportional to the field strength across the membrane.

The third branch represents ionic currents through the membrane, which arise when the ionic permeability of the membranes increases. In order to better agree with the experimental data, this model provides the possibility of the appearance of nonlinear conductance at high membrane voltages, which was observed in studies of both artificial [27] and cell membranes [28]. Following [28], where the current–voltage profile of the cell was represented by a 9th order polynomial, we represent the ion current through the membrane as

(3) $$i_{3}\left(U\right)=gU+\gamma U^{9}\;$$

where g is the linear conductance of the membrane, γ is an empirical coefficient of nonlinearity of membrane conductance.

Series resistance $R_{s}$ (access resistance) is introduced into the equivalent circuits of the model and biological membranes to account for experimental artifacts of voltage clamp measurements. The effects described by the access resistance are associated with the experimental setup, the properties of the electrodes and electrolyte, as well as the relaxation properties of the membrane itself in response to the applied voltage [19,20]. Since here we take into account the relaxation properties of the membrane in the viscoelastic circuit and the resistance of the electrodes and electrolyte is low, for simplicity, we neglect this resistance in our analysis.

Let us write the Kirchhoff equations separately for the upward (up) and downward (down) half-periods of the triangular voltage for the viscoelastic circuit.

(4) $$ri_{1,up}+{\tilde{U}}_{up}=-U_{max}+kt\;\;\;\;\;\;\;\;\;0<t<\frac{T}{2}$$

(5) $$ri_{1,down}+{\tilde{U}}_{down}=+U_{max}-kt\prime \;\;\;\;\;\;\;\;\;0<t\prime <\frac{T}{2}\;$$

The right-hand sides of Equations (4) and (5) represent the command triangular voltage supplied from the generator, ${\tilde{U}}_{up}\left(t\right),\;{\tilde{U}}_{down}\left(t\prime \right)$ are voltages across capacitance $C_{1}$ of the viscoelastic part of the membrane, causing an elastic response, for two half-cycles of the command voltage, $U_{max}$ is triangular voltage amplitude, k is sweep ramp. The voltage on the right side is proportional to the field strength in the membrane, voltage $\tilde{U}$ proportional to the electrical displacement, which, due to viscous processes, lags behind the field strength.

Substituting (1) into Equations (4) and (5), respectively, we obtain two nonlinear differential equations for ${\tilde{U}}_{up}\left(t\right)$ and ${\tilde{U}}_{down}\left(t\prime \right)$*,* corresponding to the upward and downward the command voltage ramping

(6) $$\;\;\;\;\;\;\;\;\;\;\;\;\;\;\;\frac{d{\tilde{U}}_{up}}{dt}\left(1+\beta {\tilde{U}}_{up}^{2}\right)+\frac{{\tilde{U}}_{up}}{\tau }=-\frac{U_{max}}{\tau }+\frac{kt}{\tau },\;\;\;\;\;\;\;\;\;\;\;\;\;\;\;0\leq t\leq \frac{T}{2}$$

(7) $$\frac{d{\tilde{U}}_{down}}{dt\prime }\left(1+\beta {\tilde{U}}_{down}^{2}\right)+\frac{{\tilde{U}}_{down}}{\tau }=\frac{U_{max}}{\tau }-\frac{kt\prime }{\tau },\;\;\;\;\;0\leq t\prime \leq \frac{T}{2}\;$$

where $\tau =\left(1-\varkappa \right)rC_{0}$.

Solving Equations (6) and (7) using the small parameter method (see Supplementary Material, Equations 3S–14S), we obtain expressions for the voltage across the capacitance C_1_ of the viscoelastic branch for the upward and downward half-cycles of the command triangular voltage

(8) $${\tilde{U}}_{up}\left(t\right)=\left(-U_{max}-k\tau +kt\right)+A_{up}exp\left(-\frac{t}{\tau }\right)-\beta k\tau \left[U_{max}{}^{2}+4U_{max}k\tau +5{\left(k\tau \right)}^{2}\right]+2\beta k\tau \left(U_{max}+2k\tau \right)kt-\;\beta k\tau {\left(kt\right)}^{2}\;$$

(9) $${\tilde{U}}_{down}\left(t\prime \right)=\left(U_{max}+k\tau -kt\prime \right)+A_{down}exp\left(-\frac{t\prime }{\tau }\right)+\beta k\tau \left[U_{max}{}^{2}+4U_{max}k\tau +5{\left(k\tau \right)}^{2}\right]\;-2\beta k\tau \left(U_{max}+2k\tau \right)kt\prime +\beta k\tau {\left(kt\prime \right)}^{2}\;$$

Figure 1a shows the command voltage U(t), voltages ${\tilde{U}}_{up0}\left(t\right)$ and ${\tilde{U}}_{down0}\left(t=t\prime +\frac{T}{2}\right),$ calculated by Formulas (S9) and (S10) in the zero linear approximation (β = 0) and nonlinear voltages ${\tilde{U}}_{up}\left(t\right)$ and ${\tilde{U}}_{down}\left(t=t\prime +\frac{T}{2}\right).$ Without taking into account transient processes, we can write ${\tilde{U}}_{0}\left(t\right)=U(t-\tau$*),* which is equivalent to the electrical displacement lagging behind the field strength *D(t)~E(t−τ).* Thus, the use of series resistance in the equivalent circuit describes a situation that occurs when the frequencies of electromagnetic fields correspond to the frequencies characteristic for establishing the electric polarization of a substance [29].

The currents in the viscoelastic circuit $i_{1,up}\left(t\right)$ and $i_{1,down}(t\prime )$ can be found from Equations (4) and (5), taking into account (8) and (9).

(10) $$i_{1,\;\;up}\left(t\right)=\left(1-\varkappa \right)kC_{0}\left\{1+\beta \left[U_{max}{}^{2}+4U_{max}k\tau +5{\left(k\tau \right)}^{2}\right]\right\}-2\beta kC_{0}\left(1-\varkappa \right)\left(U_{max}+2k\tau \right)kt\;+\beta kC_{0}\left(1-\varkappa \right){\left(kt\right)}^{2}-2kC_{0}\left(1-\varkappa \right)\exp\left(-\frac{t}{\tau }\right)\;\;$$

(11) $$i_{1,down}(t\prime )=-\left(1-\varkappa \right)kC_{0}\left\{1+\beta \left[U_{max}{}^{2}+4U_{max}k\tau +5{\left(k\tau \right)}^{2}\right]\right\}+2\beta kC_{0}\left(1-\varkappa \right)\left(U_{max}+2k\tau \right)kt\prime \;-\beta kC_{0}\left(1-\varkappa \right){\left(kt\prime \right)}^{2}+2kC_{0}\left(1-\varkappa \right)\exp\left(-\frac{t\prime }{\tau }\right)\;\;$$

In order to find formulas for pinched hysteresis loops, we find expression for the time inverted current response of the viscoelastic part of the membrane to the downward half-period of the command voltage (Figure 1b), substituting $t\prime =\frac{T}{2}-t$ into (11) and given that $\frac{kT}{2}=2U_{max}$*:*

(12) $$i_{1,down,inv}\left(t\right)=i_{1\;down}\left(t\prime =\frac{T}{2}-t\right)=-k\left(1-\varkappa \right)C_{0}\left\{1+\beta \left[U_{max}{}^{2}+4U_{max}k\tau +5{\left(k\tau \right)}^{2}\right]\right\}+\;2\beta kC_{0}\left(1-\varkappa \right)\left(U_{max}+2k\tau \right)\left(2U_{max}-kt\right)-\beta kC_{0}\left(1-\varkappa \right){\left(2U_{max}-kt\right)}^{2}+2kC_{0}\left(1-\varkappa \right)\exp\left(-\frac{T/2-t}{\tau }\right)\;$$

We express the time dependences of currents (10) and (12) as a function of command voltage by substituting $kt=U+U_{max}$. The currents (2), (3) are rewritten for the upward and downward half-periods: $i_{2,up}\left(U\right)=\varkappa C_{0}k,\;i_{2,down,\;inv}\left(U\right)=-\varkappa C_{0}k$, $i_{3,up}\left(U\right)=i_{3,down,\;inv}\left(U\right)=gU+\gamma U^{9}$.

Then, the total current response of the membrane to the upward and downward half-period of command voltage:

(13) $$I_{0,up}\left(U\right)=kC_{0}\left\{1+\beta \left(1-\varkappa \right)\left[U^{2}+5{\left(k\tau \right)}^{2}\right]\right\}+\left[g+\gamma U^{8}-4\beta kC_{0}\left(1-\varkappa \right)k\tau \right]U-2kC_{0}\left(1-\varkappa \right)exp\left(-\frac{U+U_{max}}{k\tau }\right)\;$$

(14) $$I_{0,down}\left(U\prime \right)=-kC_{0}\left\{1+\beta \left(1-\varkappa \right)\left[{U\prime }^{2}+5{\left(k\tau \right)}^{2}\right]\right\}+\left[g+\gamma {U\prime }^{8}-4\beta kC_{0}\left(1-\varkappa \right)k\tau \right]U\prime +2kC_{0}\left(1-\varkappa \right)exp\left(-\frac{U_{max}-U\prime }{k\tau }\right)\;$$

Total inverted current response to the downward half-period of command voltage

(15) $$I_{0,\;down,\;inv}\left(U\right)=-kC_{0}\left\{1+\beta \left(1-\varkappa \right)\left[U^{2}+5{\left(k\tau \right)}^{2}\right]\right\}+\left[g+\gamma U^{8}-4\beta kC_{0}\left(1-\varkappa \right)k\tau \right]U+2kC_{0}\left(1-\varkappa \right)exp\left(-\frac{U_{max}-U}{k\tau }\right)\;$$

The hysteresis loop equations in the C, U coordinates are given by the expressions:

(16) $$C_{up}=\frac{I_{0up}}{k}=C_{0}\left\{1+\beta \left(1-\varkappa \right)\left[U^{2}+5{\left(k\tau \right)}^{2}\right]\right\}-2\left(1\;\;\;\;\;\;\;\;\;\;\;\;\;\;\;\;\;-\varkappa \right)exp\left(-\frac{U+U_{\max}}{k\tau }\right)+\frac{1}{k}\left[g+\gamma U^{8}-4\beta kC_{0}\left(1-\varkappa \right)k\tau \right]U\;\;$$

(17) $$C_{down}=\frac{I_{0down,inv}}{-k}\;\;\;\;\;\;\;\;\;\;\;\;\;\;\;\;\;\;\;\;\;\;\;\;\;=C_{0}\left\{1+\beta \left(1-\varkappa \right)\left[U^{2}+5{\left(k\tau \right)}^{2}\right]\right\}-2\left(1\;\;\;\;\;\;\;\;\;\;\;\;\;\;\;\;\;\;\;\;\;\;\;\;\;\;\;\;\;\;\;\;-\varkappa \right)exp\left(-\frac{U+U_{\max}}{k\tau }\right)-\frac{1}{k}\left[g+\gamma U^{8}-4\beta kC_{0}\left(1-\varkappa \right)k\tau \right]U\;\;$$

### 2.2. Experimental

**Lipids and electrolytes.** Azolectin and 1,2-diphytanoyl-sn-glycero-3-phosphocholine (Avanti Polar Lipids, Alabaster) were used for the formation of planar BLM. The bulk solution contained 0.1 M KCl. The reagent was of analytical grade.

**Planar lipid bilayer membranes.** The BLMs were formed according to [30] over a 0.5 mm^2^ circular hole in a 1 mm thick wall of a Teflon chamber at room temperature of 21 ± 1 °C. The wall separated two subchambers, each filled with 2.5 mL of the same electrolyte solution. The membrane-forming solution contained 30 mg of lipids dissolved in 1 mL of n-decane. Before each experiment, the vertical wall of the Teflon chamber was covered with a thin layer of dried membrane-forming solution. Once a small droplet (~0.1 µL) of lipid solution is placed below the hole, a bilayer is formed automatically in ~10 min. The formation of the bilayer was followed by capacitance measurements. To estimate the specific capacitance of the membrane, the area of the membrane formed on the hole was determined using a microscope. The specific capacitances of the studied membranes were in the range of 3–4 nF/mm^2^.

**Nanoparticles.** To change the membrane stiffness, hydrophobic cubic CoFe_2_O_4_ nanoparticles dispersed in toluene were added to the membrane solution at a concentration of 70 μg/mL (synthesized at MISIS, Moscow, Russia). The average diagonal of the nanoparticles was 14 nm.

**Electrical measurements.** Ag-AgCl STREF1 electrodes (OHAUS Corporation, Parsippany, New Jersey, USA) were placed in both compartments of the chamber. Transmembrane currents were detected with a VA-10X amplifier (NPI Electronics GmbH, Germany) in voltage clamp mode. Currents were recorded with a sampling rate of 1 kHz in a 16-digit ADC (L-Card, Moscow, Russia). The measurements were carried out in the voltage-clamp conditions. An AKIP 3409/1 arbitrary waveform generator (PRIST, Russia) was used to apply a triangular voltage to the membrane.

**Description of the experiment.** A periodic triangular voltage of various frequencies and amplitudes was applied to the membrane in voltage clamping mode, and the current responses of the membrane were recorded. Separately, the records of the current were distinguished in response to the upward and downward half-periods of the command voltage. The current responses to downward voltage ramping were inverted over time (Figure 1b). The data were presented in the form $I_{0,up}\left(U\right),$ $I_{0,\;down,\;inv}\left(U\right)$. The experimental pinched-hysteresis loops were plotted using the curves $\frac{I_{0,up}\left(U\right)}{k}$ and $-\frac{I_{0,\;down,\;inv}\left(U\right)}{k}$ in absolute or relative capacitance units.

The measurements were carried out as follows: a triangular voltage with amplitudes of 50, 100, 130, 150, 175, 200, 225, 250, 275 and 300 mV was applied to the formed membranes; measurements were carried out at frequencies of 0.01, 0.1, 0.2, 0.35, 0.5, 0.66, 0.73, 0.8, 0.88, 1, 1.33, 1.5 and 2 Hz. For each amplitude of the triangular voltage, a current response was recorded with a duration of 20–30 periods. In each fragment, the current responses to the upward and downward half-periods of the command voltage were distinguished. To reduce the level of fluctuations at low frequencies, the signal was averaged over 30 ms. At low frequencies (<0.35 Hz), the membranes, as a rule, could not withstand high voltage; therefore, the results of measurements at the maximum amplitude before the membrane rupture were processed.

At a frequency of 0.01 Hz, two membranes were measured (triangular voltage amplitude 100 mV), at a frequency of 0.1 Hz- 2 membranes (100 mV), 1 membrane (150 mV) and 1 membrane (200 mV in the presence of particles). At a frequency of 0.2 Hz, 1 membrane was measured at amplitudes up to 200 mV, 1 membrane (150 mV) and 2 membranes (100 mV), at a frequency of 0.35 Hz—2 membranes (150 and 175 mV). At a frequency of 0.5 Hz, 4 membranes were measured at 130 mV and 2 membranes at 150 mV. A membrane was studied for which the rate of the membrane voltage sweep k = 0.8 V/s was the same for different frequencies and voltages: 0.66 Hz and 300 mV, 0.73 Hz and 275 mV, 0.8 Hz and 250 mV, 0.88 Hz and 225 mV, 1.33 Hz and 150 mV. At a frequency of 1 Hz, more than 20 membranes at 50 mV and 100 mV, 4 membranes at 150 mV and 14 membranes at 200 mV were recorded and processed. The current responses to a triangular voltage with an amplitude of 50 mV showed no nonlinearity or hysteresis, but we used them for transient analysis (in these cases, the signal was not averaged). At a frequency of 1.5 Hz, the current responses of the membrane were recorded at amplitudes up to 200 mV. At a frequency of 2 Hz, the currents of 4 membranes were recorded at command voltage amplitudes of up to 300 mV and of 6 membranes of up to 200 mV. The current responses to a triangular voltage with a frequency of 0.1 Hz and 1 Hz and an amplitude of 200 mV were studied for two membranes in the presence of nanoparticles.

## 3. Results

### 3.1. Calculation of the Parameters of the Equivalent Circuit According to Experimental Results

Consider the half-sum and half-difference of the current response to the upward (13) and inverted downward (15) half-cycles of command voltage at $-U_{\max}<U<U_{\max}$.

(18) $$\frac{1}{2}\left[I_{0,up}\left(U\right)-I_{0,\;down,\;inv}\left(U\right)\right]=kC_{0}\left\{1+\beta_{exp}\left[U^{2}+5{\left(k\tau \right)}^{2}\right]\right\}-kC_{0}\left(1-\varkappa \right)\left[\exp\left(-\frac{U+U_{max}}{k\tau }\right)+\exp\left(-\frac{U_{max}-U}{k\tau }\;\right)\right]\;$$

(19) $$\frac{1}{2}\left[I_{0,up}\left(U\right)+I_{0,\;down,\;inv}\left(U\right)\right]=\left(g+\gamma U^{8}-4k\beta_{exp}C_{0}k\tau \right)U-kC_{0}\left(1-\varkappa \right)\left[\exp\left(-\frac{U+U_{max}}{k\tau }\right)-\exp\left(-\frac{U_{max}-U}{k\tau }\right)\right]\;$$

As can be seen from these formulas, without taking into account the exponents, Formula (18) corresponds to the empirical formula for the current through the nonlinear capacitance of the membrane with $\beta_{exp}=\beta (1-\varkappa )$ and $C_{exp0}=C_{0}\left[1+5\beta {\left(k\tau \right)}^{2}\right]$ with a small correction associated with a viscoelastic part. Thus, jointly using the experimental current responses to the upward and downward half-cycles of the triangular voltage, it is possible to unambiguously determine the experimental parameters $C_{exp0}{\;\mathrm{and}\;\beta }_{exp}$. It should be noted that the idea of sharing current response data for two half-cycles of a triangular voltage (a paired-ramp protocol) was used in [31,32,33,34] to measure the capacitance of the model bilayer membranes and cells. In these works, to calculate capacitance, the half-difference of the average values of the currents measured in a certain region in the middle of the half-cycles of the command triangular voltage was used.

**Figure 3:**
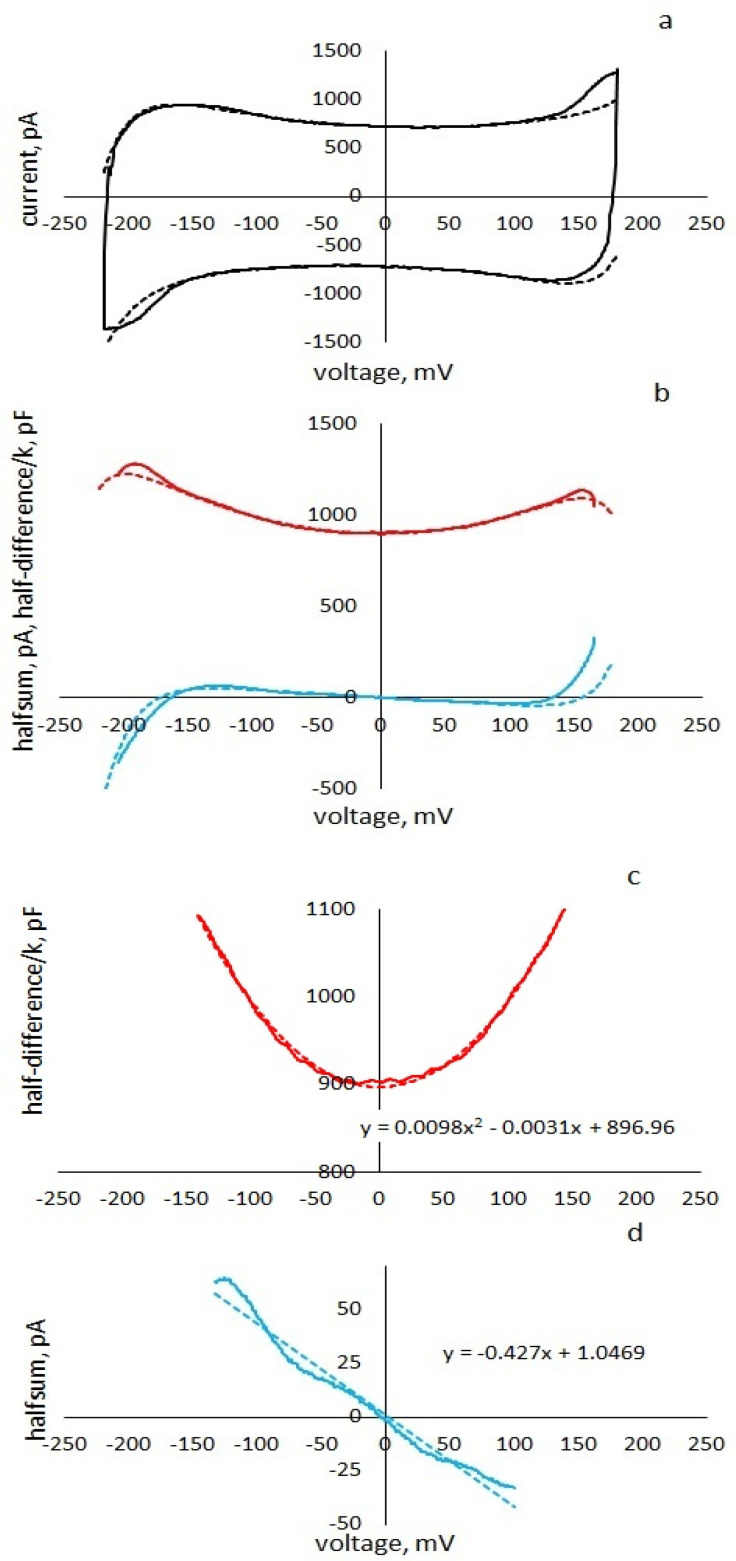
Experimental and theoretical curves of current responses of an azolectin membrane in 0.1 M KCl, to a triangular voltage with an amplitude 200 mV, a frequency 1 Hz (solid curves—experiment, dotted curves—calculation by Formulas (13), (15), (18) and (19): (**a**)—cyclic current–voltage characteristic of the membrane; (**b**)—half-difference/k—upper curve, half-sum—lower; (**c**)—fragment of the capacitance curve and its parabolic fit, (**d**)—fragment of apparent negative conductance and its linear fit.

Figure 3a shows the experimental cyclic current–voltage characteristic, the upper branch of which is the experimental response of the membrane to increasing voltage; the lower branch is the temporal inversion of the current response of the membrane to decreasing voltage.

Figure 3b shows the half-difference of the upper and lower branches of the cyclic current–voltage characteristic divided by k (in pF) at the top and the half-sum (in pA) at the bottom. The value of the parameter ϰ = 0.2, which determines the proportion of the viscoelastic part of the membrane, is selected by the point separating the two exponents of transient processes in response to a change in the sign of the slope of the triangular voltage. Figure 3c shows the upper curve in Figure 3b in the region of low voltages and the parabolic fit of this fragment. Comparison with Formula (18) makes it possible to uniquely determine the parameters: $C_{0}$ = 897 pF, $\beta_{exp}$ = 11 1/V^2^.

Formula (19), without taking into account transient processes, can be interpreted as the membrane conduction current. It can be seen from the formula that, according to the experimental data, only the value of the apparent conductance can be unambiguously determined as the coefficient at U in Formula (13):

(20) $$g_{app}=g-4k\beta_{exp}C_{0}k\tau \;$$

The first component of apparent conductance is the ionic conductance of the membrane. The second component is negative insertion conductance, the appearance of which is associated with the combined action of the nonlinearity and viscoelasticity of the membrane’s response to the applied voltage. The values of the parameters *τ* = 19 ms, *g* = 0.05 nS are found from the best match of the shape of the experimental curve (Figure 3a) with the calculation by Formulas (13) and (15) and the trend of the experimental half-sum (Figure 3d) with Formula (19). Theoretical curves, calculated by Formulas (13), (15), (18) and (19) with these parameters are shown in Figure 3 (dotted lines).

As can be seen from Figure 3a,b at high applied voltages |U|> 150 mV, the current sharply increases and the apparent conductance changes sign. In this voltage range, the conductance sharply increases and, possibly, the coefficient of nonlinearity of the capacitance decreases. For better agreement between the experimental and theoretical curves, we take into account the possibility of the appearance of nonlinear conductance at high voltages [27,28]. We approximate the conductance current by a ninth-degree polynomial (3) with the parameter γ = 0.5 10^−18^ nS/mV^8^. It should be noted that we only indicated the trend of current growth at high voltages but did not set the problem and could not adequately describe the experimental nonlinear conductance, since high currents were often cut off by the amplifier.

### 3.2. Parameter ϰ and Transients

As can be seen from Figure 3a, the initial edge of the current curve is determined by the transients that occur in response to the voltage jump $\tilde{U}$ when the sign of the slope of the triangular voltage is reversed.

**Figure 4:**
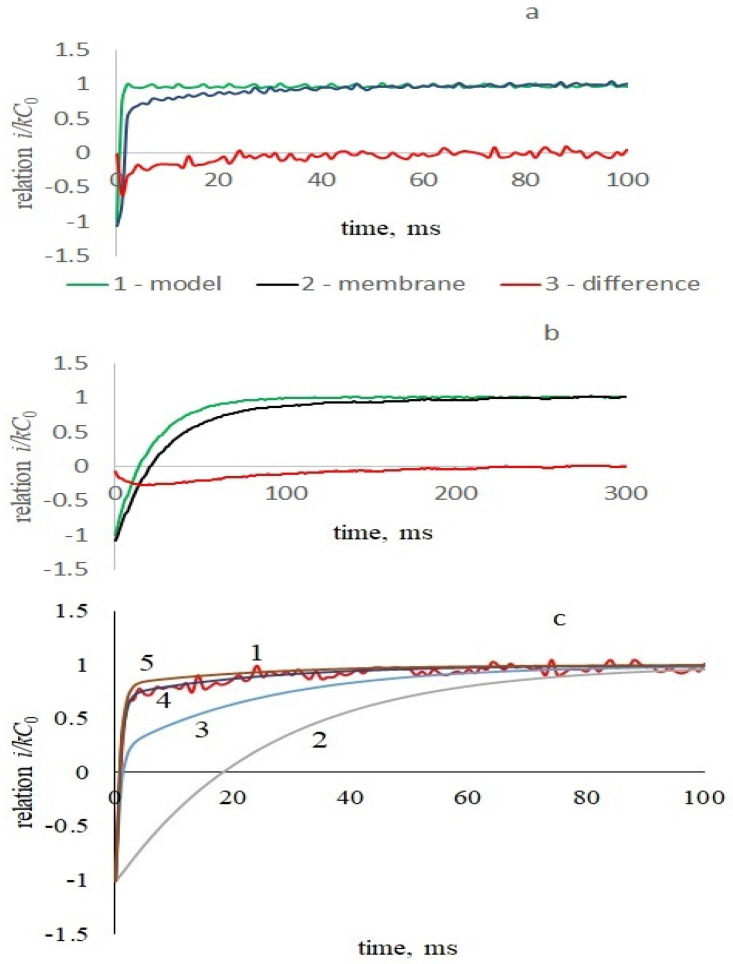
The initial phase of the jump in the amplifier output signal in response to a change in the slope of the triangular command voltage with an amplitude of 50 mV and a frequency of 2 Hz for model (1) and a membrane of azolectin in 0.1 M KCl (2), as well as their difference (3). The ordinate is the ratio $i/kC_{0}$ of the output current *i* to the steady-state capacitive current $kC_{0}$, the abscissa is the time since the beginning of the growing part of the command voltage. (**a**)—feedback resistance of the amplifier is *R_out_* = 100 MΩ, model capacitance is 3.14 nF and resistance is 680 MΩ, *τ* = 0.8 ms. (**b**)—the feedback resistance of the amplifier is 5 GΩ, the leading edge does not depend on the capacitance of the breadboard (measured for capacities of 1 and 1.89 nF, the curves in relative units coincide), there was no resistance in the model, *τ* = 21 ms. (**c**)—calculated curves for different values of 1—ϰ: 1 (2), 0.4 (3), 0.15 (4), 0.1 (5), 1—experimental.

In some works [27,33], when analyzing the current response to the command voltage in the form of a voltage step, relaxation processes are described by the sums of two exponentials. The exponent with a shorter relaxation time is associated with the influence of the setting. To refine the model and clarify the nature of the exponentials, we compared the current response of the membrane to a periodic triangular voltage with that of a model consisting of capacitance and resistance in parallel or only capacitance. Figure 4 shows the results for two amplifiers with feedback resistances of 100 MΩ (a) and 5 GΩ (b).

Figure 4a,b show that the model’s initial current response is determined by the amplifier’s feedback circuit and is independent of the capacitance in the amplifier’s input circuit. The difference between the current responses of the model and the membrane shows the exponential response of the membrane, strongly distorted by the amplifier with a large time constant (Figure 4b). To refine the model, we take into account the feedback loop of the amplifier (Figure 2). The voltage at the output of the amplifier $U_{out}$ corresponding to the current in the input circuit of the membrane $I_{0}$, determined for the upward half-cycles of the command voltage by (13) for an ideal amplifier, is described by the linear differential equation:

(21) $$\frac{dU_{out}}{dt}+\frac{U_{out}}{\tau_{out}}=\frac{I_{0}R_{out}}{\tau_{out}}$$

where $\tau_{out}=R_{out}C_{out}$; $R_{out}$ is the resistance in the feedback circuit of the amplifier, $C_{out}$ is the parasitic capacitance.

With a command voltage amplitude of 50 mV, the nonlinearities of both conductance and capacitance can be neglected; therefore, assuming β = 0 and γ = 0 and, for simplicity, g = 0 in Formula (13), we obtain:

(22) $$I_{0}=kC_{0}-2\left(1-\varkappa \right)kC_{0}\exp\left(-\frac{t}{\tau }\right)$$

Substituting (22) into Equation (21) and solving it, we find the output current of the amplifier

(23) $$\frac{U_{up,out}\left(t\right)}{R_{out}}=kC_{0}-\left(1-\varkappa \right)\frac{2kC_{0}}{1-\tau_{out}/\tau }\left[\exp\left(-\frac{t}{\tau }\right)-\exp\left(-\frac{t}{\tau_{out}}\right)\right]-\;2kC_{0}\exp\left(-\frac{t}{\tau_{out}}\right)$$

For $\tau_{out}/\tau \ll 1$ Formula (23) can be rewritten in the form

$$\frac{U_{up,out}\left(t\right)}{R_{out}}=kC_{0}-2\left(1-\varkappa \right)kC_{0}\exp\left(-\frac{t}{\tau }\right)-2\varkappa kC_{0}\exp\left(-\frac{t}{\tau_{out}}\right)$$

Figure 4c depicts the calculated initial current of the membrane response to the upward cycle of command voltage for various values of the proportion of the viscous elastic part in membrane 1 − *ϰ*. Thus, the presence of purely elastic and viscoelastic branches in the equivalent circuit makes it possible to adequately describe the transient processes in the current response of the membrane, which consists of two exponents. A value of 1 − *ϰ* in the range of 0.1–0.2 is suitable for all considered experimental data.

### 3.3. Dynamical Responses and Pinched Hysteresis

To investigate the membrane memcapacitance, we plotted both experimental and calculated hysteresis loops of the dynamic capacitance versus voltage (*C-U*). Pinched-hysteresis loops are described in Formulas (16) and (17). The width of the hysteresis loop in relative capacitance units is determined by the formula:

(24) $$\frac{C_{up}-C_{down}}{C_{0}}=\left(\frac{g}{kC_{0}}-4\beta_{exp}k\tau \right)U\;$$

It is proportional to the apparent differential negative conductance and depends on the sum of the values of the ionic conductance of the membrane and the insertion negative conductance. Insertion negative conductance depends on the nonlinear capacitance characteristics, the rate of growth of the triangular voltage and the parameter characterizing the delay time of the membrane polarization from the electric field applied.

As can be seen from Formula (24), the width of the pinched hysteresis loops depends on the parameters $C_{0},\;\beta$ and τ, as well as the slope of the triangular voltage k.

**Figure 5:**
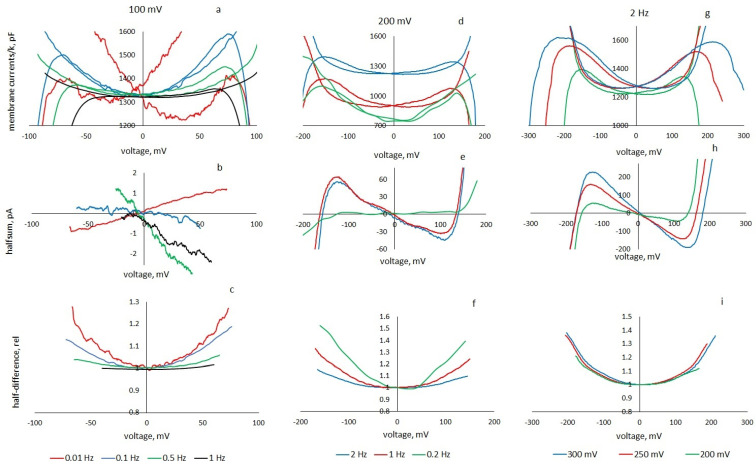
Pinched-hysteresis loops (**a**,**d**,**g**), negative apparent conductance (**b**,**e**,**h**), capacitance curves (**e**,**f**,**i**) showing coefficient β values for membranes of azolectin, 0.1 M KCl depending on the characteristics of the triangular voltage. Left column (**a**–**c**): triangular voltage amplitude 100 mV, frequencies 0.01, 0.1, 0.5 and 1 Hz (*β* = 4, 11, 29, 47 V^−2^). Middle column (**d**–**f**): triangular voltage amplitude 200 mV, frequency 2, 1 and 0.2 Hz. Right column (**g**–**i**): frequency 2 Hz, triangular voltage amplitude 200, 250 and 300 mV.

A set of representative results is presented in Figure 5. As can be seen from the left column, in the studied frequency range, the parameters of the equivalent circuit, the membrane nonlinearity, and the loop width also strongly depend on the frequency of the periodic command signal. As the frequency decreases, *k* decreases, but the nonlinearity coefficient β increases monotonically. The loop width proportional to the apparent negative conductance is maximum at an intermediate frequency of 0.5 Hz. At a frequency of 0.01 Hz, the hysteresis disappears and the apparent conductance becomes positive. At a frequency of 1 Hz, the loop decreases; at 2 Hz, the current response of the membrane corresponds to the current response of a linear capacitor. These frequency dependences are related to the characteristic times of viscoelastic processes responsible for the delay of the true field from the applied and dynamic capacitive response to a periodic triangular voltage. At the amplitude of the triangular voltage 200 mV (Figure 5, middle column), the width of the pinched hysteresis loops also strongly depends on the frequency of the command voltage. At frequencies of 1 and 2 Hz, the apparent conductance is almost the same; at a frequency of 0.2 Hz, the hysteresis disappears, and the apparent conductance becomes positive. Unfortunately, at low frequencies, the membranes cannot withstand high voltage and often break. An increase in amplitude (Figure 5, right column) at a constant frequency of 2 Hz leads to an increase in apparent conductance and loop width in proportion to increasing *k.* The nonlinearity of the capacitance practically does not change. The loop width and, accordingly, the insertion conductance increase proportionally to *k*.

**Figure 6:**
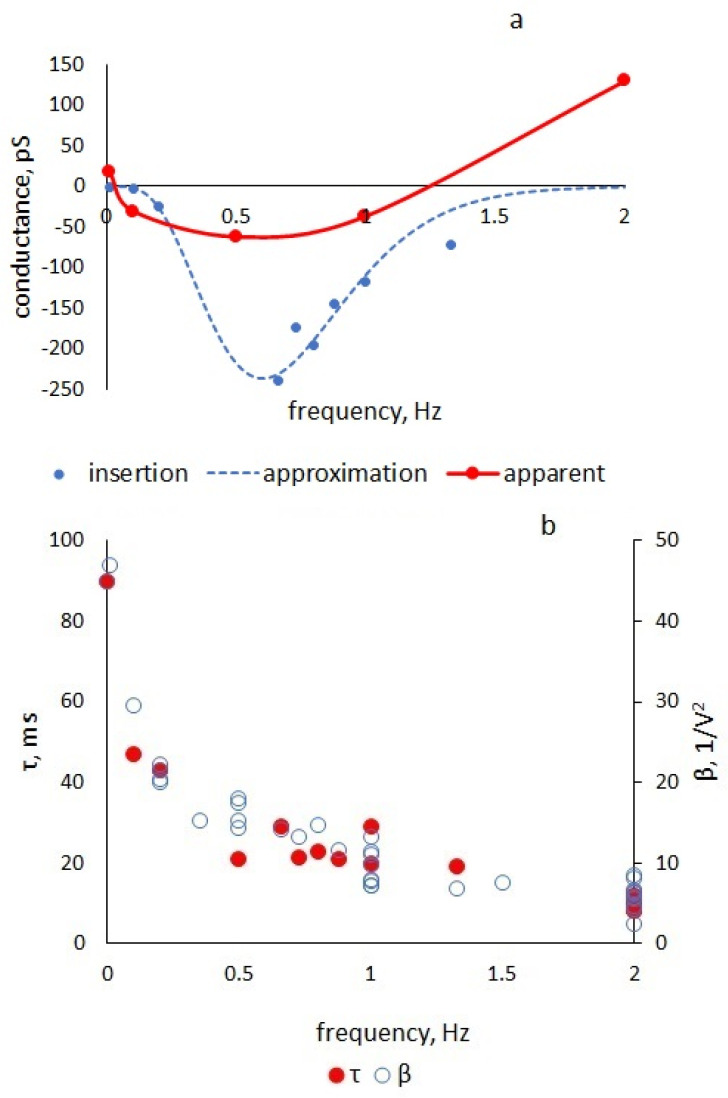
(**a**): Dependence of apparent and insertion conductance calculated by Formula (20) with parameters *β* and *τ* calculated from experimental data on triangular voltage frequency. The calculation did not take into account the ionic conductance of the membranes (*g* = 0). (**b**): Dependence of the values of *β* and *τ* calculated from experimental data on the frequency of the triangular voltage.

Figure 6a shows the dependence of the apparent conductance of the membrane on the frequency of a triangular voltage with an amplitude of 100 mV, as determined from the data in Figure 5c. The same figure shows the insertion conduction calculated by Formula (20) with the parameters shown in Figure 6b. The frequency dependence of the nonlinearity coefficient is determined as a calculated parameter from the experimental half-difference. Figure 6b shows the values of the nonlinearity coefficients for various frequencies and amplitudes of the triangular voltage. The same figure shows the frequency dependence of parameter *τ*, chosen from the coincidence of the experimental and calculated current responses to a triangular voltage of different frequencies and the slope of the apparent negative conductance.

**Figure 7:**
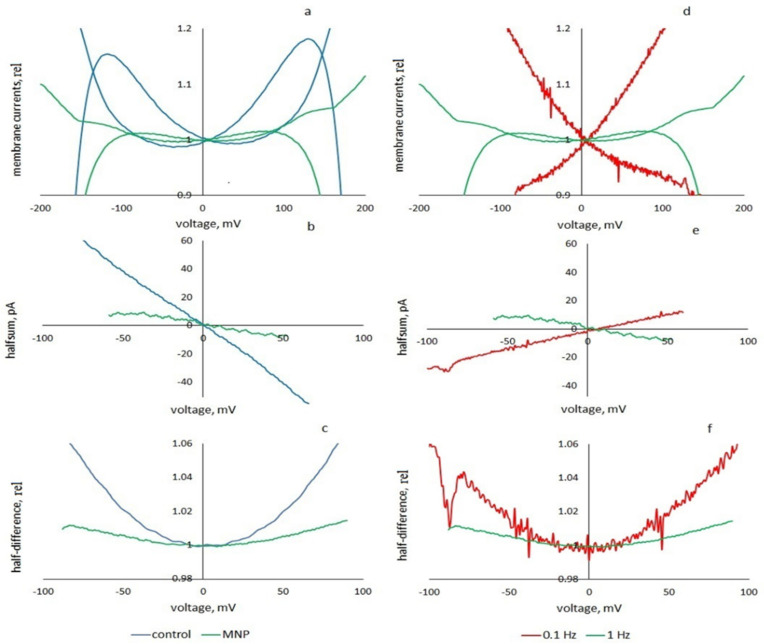
The effect of cobalt ferrite particles on the viscoelastic current responses of the membrane to a triangular voltage with an amplitude of 200 mV: hysteresis loops (**a**,**d**), apparent conductance currents (half-sums) (**b**,**e**), and nonlinear capacitance dependences (half-differences of experimental curves in relative capacitance units) (**c**,**f**). On the left—the effect of nanoparticles: the control and results are presented when particles are added, the frequency is 1 Hz; on the right—the influence of the signal frequency on the characteristics of the membrane with particles. The data are shown for frequencies of 1 and 0.1 Hz.

In [35], an increase in BLM permeability in the presence of nanoparticles was shown. Here, we decided to determine whether the current response of the membrane to triangular voltage and the properties of the membrane change when particles are added. Figure 7 shows the change in the parameters of the nonlinear dynamic characteristics of the membrane when hydrophobic nanoparticles of cobalt ferrite are added to the membrane solution. As can be seen from Figure 7, the presence of particles changes the nonlinear dynamic characteristics of the membrane: it leads to a noticeable decrease in the nonlinearity coefficient (from 8.3 to 2 V^−2^), a decrease in the width of the hysteresis loop and an apparent negative conductance. The right side of the figure shows the effect of frequency on the characteristics of a membrane with particles—as the frequency decreases from 1 to 0.1 Hz, the nonlinearity coefficient increases from 2 to 6 V^−2^, while the hysteresis disappears, and the apparent conductance is positive. Compared to particle-free membranes, the increase in nonlinearity with decreasing frequency is approximately the same, but at a lower level.

## 4. Discussion

All putative processes affecting the nonlinear dependence of capacitance on voltage (electrostriction, solvent redistribution, thermal fluctuations) do not depend on the direction of the field and are approximated by an even degree polynomial. These effects are dissimilar in physical origins and differ in the intensity of the action and different time scales [1,2,3,4,5,10,25,26]. In the experiments we describe, which illustrate the adequacy of our nonlinear mathematical model, the main reason for the nonlinear response of the membrane to a triangular voltage at frequencies of 0.1–2 Hz and an amplitude of up to 300 mV is a decrease in the thickness and, possibly, an increase in the area of the bilayer. These changes in geometry arise due to the redistribution of the solvent between the microlenses and the boundary and the electrostrictive compression of the monolayers.

It is known that bilayer lipid membranes with a solvent allow the electric field to create large changes in geometry compared to “dry” membranes [1,7,11] and, accordingly, give well-interpreted current responses to a periodic electrical stimulus. Although the solvent is not part of biological membranes, an artificial membrane can serve as a model for studying the influence of the characteristics of a periodic electrical field (amplitude, frequency, shape) on the nonlinear electrical characteristics of the membrane and their correlation with the properties of the membrane under various conditions.

In an equivalent circuit following the logic of work [23] related to modeling viscoelastic dielectrics, we have divided the capacitive current of the membrane into two parts. The first part is the current through the purely elastic linear part of the membrane. The second part is the current through the viscoelastic nonlinear part, the polarization of which lags behind the command voltage. In the proposed equivalent circuit, this lag is provided by a resistance in series with the viscoelastic part of the capacitance. As a result of solving a nonlinear differential equation using the small parameter method, we obtain an analytical dependence of the current response of a membrane with quadratic capacitance nonlinearity to a periodic triangular voltage on time and membrane voltage. Already, the first approximation of the method makes it possible to adequately describe the main effects observed in the experiment. A comparison of the calculation with the experiment makes it possible to determine the parameters of the equivalent circuit.

The nonlinearity coefficient *β* is maximum in the static mode and decreases with increasing frequency due to a decrease in the number of processes that have time to follow changes in the alternating field. For example, at a frequency of 125 Hz [10], the coefficient of nonlinearity for the capacitance of BLM from azolectin in decane was 0.6 V^−2^, while in our measurements, it varied from 47 to 5 V^−2^ in the range of 0.01–2 Hz (Figure 6).

The series resistance in our equivalent circuit is responsible for the relaxation of the molecular processes occurring in the membrane under the action of an alternating electric field. An analysis of the equation solution in comparison with the experiment shows that the value of the equivalent series resistance enters the solution only through parameter τ, which characterizes the membrane polarization delay with respect to the applied voltage. This parameter, as well as the capacitance nonlinearity coefficient β, depend on the frequency of the periodic command voltage (Figure 6) and on the relaxation times of capacitive processes occurring in the membrane under the action of an electric field. The frequency range in which we observe changes in the parameters corresponds to the relaxation times of the solvent redistribution and expansion of the plato border 5 ms–5 s [4,5,24,25,26]. We use an empirical formula for the nonlinear capacitance in our model without considering the physical nature of the processes leading to nonlinearity. However, we believe that in our particular experimental situation τ depends on the viscosity of the BLM solvent, which is confirmed by studies of the capacitance characteristics for membranes with various solvents [11].

A similarly shaped current response to a triangular voltage of higher frequencies and, accordingly, associated with other physical processes with corresponding relaxation times was discussed in [10]. To take into account time dependences, capacitance was introduced into the model, which depends on time according to an exponential law in the same way as in [22]. Since the processes preventing an instantaneous change in the polarization of the membrane under the action of voltage are associated with energy losses, it seems more physically adequate to use an equivalent circuit with a series of active resistance depending on the frequency.

The existence of hysteresis phenomena in the current response of a nonlinear membrane to a periodic triangular voltage was noted in early works [5,6]. These phenomena are also discussed in works where DIB is used as a biological membrane model and dynamic capacitive currents in membranes with different solvents are studied [7,8,11]. It was shown in [11] that the existence of hysteresis is associated with voltage-dependent changes in the bilayer geometry, which depend on the viscosity of the solvent (decane and hexadecane were compared), temperature and the frequency of the command sinusoidal voltage.

In this context, in [11], it is proposed to consider DIB as the only variant of the physical implementation of a memcapacitor with a geometry that changes under the action of an electric field, where the effective distance between the capacitor plates or its area changes in some way under the action of the applied voltage.

The authors believe that the presence of so-called pinched hysteresis in the charge–voltage and capacitance–voltage coordinates makes it possible to assert that DIB with a solvent is a realistic and physically justified model for passive memcapacitance in the understanding of [12,13,14]. It is hypothesized that the non-conductive lipid bilayer of cells (without ion channels) may also exhibit capacitive memory driven by voltage-dependent changes of the dielectric, dominated by the hydrophobic core of the bilayer. In DIB experiments, pinched hysteresis in the capacitance–voltage planes results from dynamic changes in interfacial area and hydrophobic thickness, each of which is nonlinearly dependent on voltage. They believe that studies of bilayer lipid membranes can predict new classes of biomimetic, low-power memelements based on soft, organic materials and biomolecules, which, in turn, aid in exploring capacitive memory and susceptibility in neuronal membranes.

We have shown that in the formula for the BLM current response to an applied triangular periodic voltage, due to a combination of nonlinearity and polarization time delays, insertion negative conductance appears, the effect of which is limited by the ion permeability of the membranes. The total apparent conductance is a measure of pinched hysteresis. When the negative insertion conductance is small or compensated for by the ionic conductance of the membranes, the hysteresis and, correspondingly, the memcapacitance properties disappear. Our model allows us to obtain analytical dependences of the curves forming hysteresis loops (expressions (16), (17)) and to analyze the causes and features of these loops.

Analyzing expressions (8, 9), we see that due to the presence of a series resistance in the viscous branch of the equivalent circuit, the voltage $\tilde{U}$ lags from the command triangular voltage U, proportional to the field strength (Figure 1). Because of this, hysteresis appears on the experimental and theoretical curves $I_{0}=f\left(U\right)$ in the presence of capacitance nonlinearity. The general form of the formulas for the current of both half-cycles (13), (14) corresponds to the condition memcapacitance $\;I_{c}=A-BU+DU^{2\;}$ [17] at $g<4k\beta_{exp}C_{0}k\tau$.

**Figure 8:**
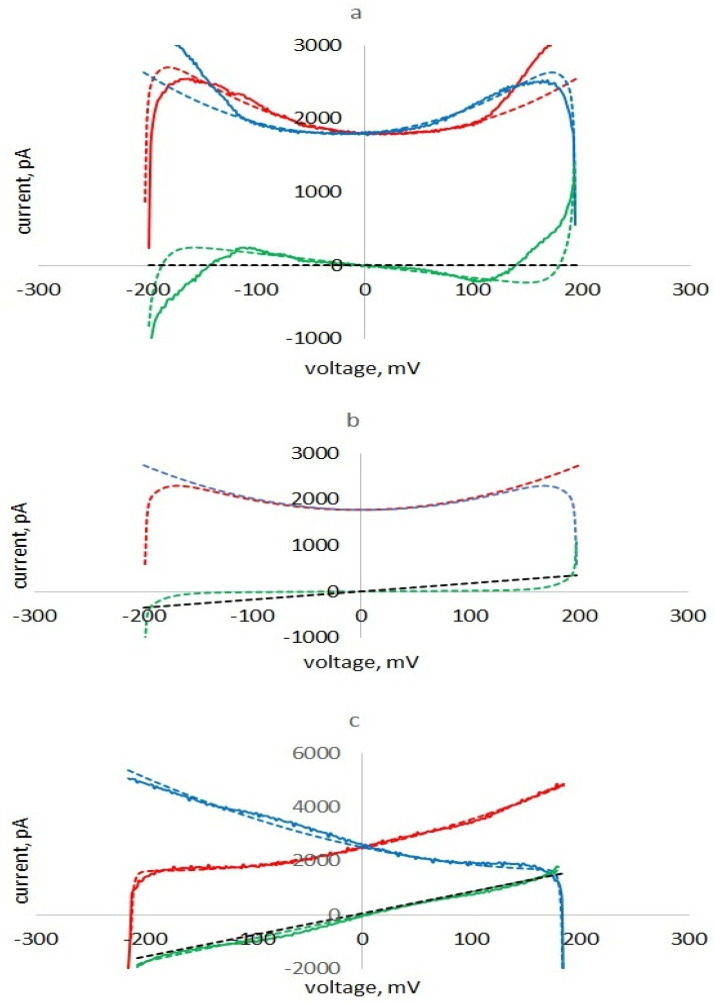
Experimental (solid) and calculated (dotted lines) hysteresis curves $I_{0,\mathrm{up}}$ (red), $I_{0,\;\mathrm{down},\;\mathrm{inv}}$ (blue) and their halfsum (green) and calculated ionic currents (dotted black lines) for three cases: with negative (**a**), zero (**b**) and positive (**c**) apparent conductance. Experimental curves—current responses of the azolectin membrane in 0.1 M KCl with low (**a**) and high (**c**) conductance to a triangular voltage with a frequency of 2 Hz and an amplitude of 200 mV. Equivalent circuit parameters: C_0_ = $1120pF;\;\tau =10\;ms;\beta =15\;V^{-2};f=2\;Hz;U_{max}=200\;mV$; γ = 0; *g* = 0 (**a**), 1.72 *nS* (**b**), 8 *nS* (**c**).

The presence in the current of a term with a negative sign, proportional to the voltage, is determined by the nonlinearity of the capacitance and the shift in the minimum of the capacitive current relative to zero of the command voltage associated with the delay in the polarization of the membrane. As a result (see Figure 8a), the part of the half cycle under the left falling part of the parabola (the capacitor gives energy to the circuit) is greater than the part when the capacitor is charged (energy is taken). The difference between these energies is proportional to the negative insertion conductance, which reduces the losses in the membrane. The calculated current curves of two half-cycles, forming pinched hysteresis loops, are shown in Figure 8a–c for different values of the membrane ionic conductance.

When $g+\gamma U^{8}<4\beta kC_{0}k\tau ,$ the term in expression (13) proportional to U is negative and the expression corresponds to the memcapacitance condition (Figure 8a). The current minima in the upward and downward half cycles of the applied voltage are shifted to the right (see Figure 8a). The energy released with a decrease in capacitance is greater than the energy taken from the generator with an increase in capacitance; the apparent conductance is negative, and hysteresis is observed.

When $g+\gamma U^{8}=4\beta kC_{0}k\tau$ the current minima in the upward and downward half cycles of applied voltage coincide and are observed at U = 0 (see Figure 8b). The energy released when the capacitance decreases is equal to the energy taken from the generator with an increase in capacitance; the apparent conductance and the loop width are zero.

When $g+\gamma U^{8}>4\beta kC_{0}k\tau$ the term in expression (13) proportional to U is positive and hysteresis is not observed (Figure 8c).

Thus, the observed pinched hysteresis is associated with nonlinear parametric capacitance effects and is limited by membrane ionic conductance. In Figure 8a,c, for comparison, experimental curves for membranes with low (a) and high (c) ionic conductance are shown.

Let us calculate the work of the source in a nonlinearly viscous system (without taking into account transient processes), integrating over time over the period the product of the total current and the command voltage. Assuming in Formulas (13) and (14) $\gamma =0,\;\beta_{exp}=\left(1-\varkappa \right)\beta$, $U=-U_{max}+kt;U\prime =U_{max}-kt$, we get

(25) $$W=\frac{T}{3}U_{max}^{2}\left(g-4\beta kC_{0}k\tau \right)$$

Under the conditions of the existence of pinched hysteresis $g<4\beta kC_{0}k\tau$ the work is negative and the memcapacitance process is active.

Thus, the combination of nonlinear geometrical changes in the membrane under the action of an electric field and a delay in the polarization of the membrane due to viscous processes does not lead to energy losses, for example, in a ferroelectric, but leads to the appearance of an insertion negative conductance. The importance of the shape of the curve of the nonlinear dependence of capacitance on voltage for viscoelastic effects was noted in [23] when analyzing a dielectric elastomer. Nonlinear charge–voltage characteristics for a membrane $q=C_{0}U+\frac{\beta }{3}C_{0}U^{3}$ and a ferroelectric $U=\frac{1}{C_{0}}q+\frac{1}{C_{0}}\frac{\beta }{3}q^{3}$ are inverse functions.

The difference in the energetics of the processes can be shown by solving for a ferroelectric a similar nonlinear differential equation for *q*, (see Supplementary Material, Equations 15S–20S). Current response to the upward half-cycle of the triangular voltage

(26) $$I_{0up}=kC_{0}\left(1-\beta_{exp}C_{0}^{2}\left(U^{2}+5{\left(k\tau \right)}^{2}\right)\right)+\left(g+4\beta_{exp}kC_{0}^{3}k\tau \right)U\;$$

**Figure 9:**
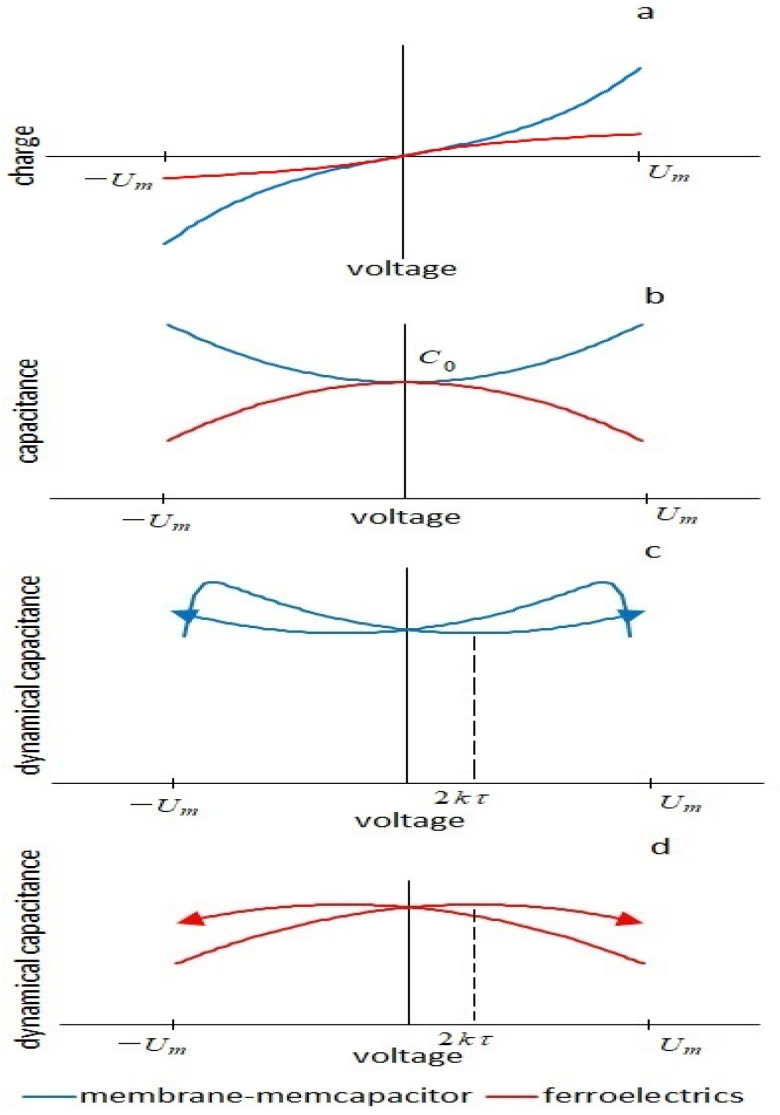
Characteristics of membrane-memcapacitor (blue) and ferroelectrics (red): (**a**)—charge–voltage characteristics, (**b**)—dependences of the nonlinear capacitance on voltage. For the membrane in the absence of viscous effects in the first quarter of the period, the capacitor is discharged, the source work is negative, in the second quarter it is charged, the source work is positive, the source work for the half-cycle is zero, and the system is neutral. For a ferroelectric in the first quarter—charging, in the second—discharging, the system is neutral. (**c**)—dependence of the dynamic capacitance of the membrane (of the ferroelectric) on voltage. Due to the delay in polarization, part of the half-cycle of the triangular voltage, when the capacitor is discharged, increases; as a result, there is a negative insertion conductance. Delaying polarization with this type of nonlinearity reduces losses and results in pinched hysteresis. The system is active. (**d**)—dependence of the dynamic capacitance of the ferroelectric on voltage. Due to the delay in polarization, the part of the half-cycle of the triangular voltage, when the capacitor is charged, increases; as a result—there is a positive insertion conductance. Delaying polarization with this type of nonlinearity increases losses and does not show pinched hysteresis. The system is passive.

Comparing this expression with Formula (13), one can see that, unlike the capacitance of the membrane, the capacitance of a ferroelectric decreases with increasing voltage (in the first approximation, along a parabola). This leads to the fact that the insertion conductance in a circuit with ferroelectrics is positive and increases the losses in the system. There is no pinched hysteresis in such a system. Thus, capacitive pinched hysteresis requires a combination of nonlinear capacitance vs. voltage at which the capacitance increases with an increase in the electric field and a delay in the process of polarization of the dielectric, leading to an increase in the fraction of energy given off by the capacitor to the circuit during the upward half-cycle of the triangular voltage. Qualitatively, the energetics of the processes are shown in Figure 9.

## 5. Conclusions

We propose an equivalent circuit and mathematical framework for analyzing the dynamic current response of a lipid bilayer membrane as an externally controlled memcapacitance. We apply our equivalent circuit to obtain analytical solutions for the current response of a membrane to a triangular voltage without the need for numerical simulations. Our explicit solutions and their comparison with the experimental data allow us to identify the parameters of the equivalent circuit. These parameters control the memcapacitor behavior of the membrane and, consequently, the amount of hysteresis in the C-U characteristic.

We quantify the memcapacitance hysteresis in terms of the work done by the control signal. We use the resulting analytical solutions to show that device hysteresis depends on the negative conductance inserted by the non-linear capacitor, for which we obtain explicit formulas in terms of the equivalent circuit parameters and characteristics of the input signal.

The effects of the memcapacitance of membranes depend on the coefficient of nonlinearity of the capacitance and the parameter τ, which is associated with the relaxation processes of capacitance change under the action of voltage. These processes can be of a different nature and manifest themselves in different frequency ranges, depending on the properties of the membrane (lipid, additives affecting the hydrophobic part of the membrane). Memory effects may disappear with an increase in the ion permeability of the membranes.

## Data Availability

Not applicable.

## Supplementary Materials

The following supporting information can be downloaded at: https://www.mdpi.com/article/10.3390/membranes13010097/s1. S1: Solution of a Nonlinear Differential Equation for the Equivalent Circuit of a Membrane as a Viscoelastic Dielectric. S2: Solution of the differential equation for a ferroelectric.
