# Supplementary material for: Bilayer Lipid Membrane as Memcapacitance: Capacitance–Voltage Pinched Hysteresis and Negative Insertion Conductance
Source: Membranes (Basel). 2023 Jan 11;13(1):97. doi: 10.3390/membranes13010097 (PMC9861822; doi:10.3390/membranes13010097)
Supplement: Supplementary file 1 [file membranes-13-00097-s001.zip › membranes-2122356-SI.pdf]

Supplementary materials

1S. Solution of a Nonlinear Differential Equation for the Equivalent Circuit of a Membrane as a Viscoelastic Dielectric

We write nonlinear equations for two half-cycles of a triangular voltage

$$\frac{d\tilde{U}_{up}}{dt}(1+\beta\tilde{U}_{up}^2)+\frac{\tilde{U}_{up}}{\tau}=-\frac{U_{max}}{\tau}+\frac{kt}{\tau},\ 0\le t\le\frac{T}{2} \quad (S1)$$

$$\frac{d\tilde{U}_{down}}{dt'}(1+\beta\tilde{U}_{down}^2)+\frac{\tilde{U}_{down}}{\tau}=\frac{U_{max}}{\tau}-\frac{kt'}{\tau},\ 0\le t'\le\frac{T}{2}, \quad (S2)$$

where $\tau=(1-\varkappa)rC_0$.

We will solve nonlinear equations (S1) and (S2) using the small parameter method. Let us introduce μ ($0<\mu\ll 1$) – a positive small parameter and considering the coefficient of nonlinearity to be small and proportional to μ , we will look for the solution of equations (S1) and (S2) in the form of a series in powers of the small parameter:

$$\tilde{U}_{up}=\tilde{U}_{up0}+\mu\tilde{U}_{up1}+\cdots \quad (S3)$$

$$\tilde{U}_{down}=\tilde{U}_{down0}+\mu\tilde{U}_{down1}+\cdots. \quad (S4)$$

Substituting (S3), (S4) into equations (S1) and (S2) and equating terms with the same μ degree, we obtain two linear differential equations that determine the voltage $\tilde{U}$ for each half-cycle of the command voltage with accuracy up to the first order of smallness

$$\frac{d\tilde{U}_{up0}}{dt}+\frac{\tilde{U}_{up0}}{\tau}=-\frac{U_{max}}{\tau}+\frac{kt}{\tau} \quad (S5)$$

$$\frac{d\tilde{U}_{up1}}{dt}+\frac{\tilde{U}_{up1}}{\tau}=-\beta\tilde{U}_{up0}^2\frac{d\tilde{U}_{up0}}{dt} \quad (S6)$$

$$\frac{d\tilde{U}_{down0}}{dt'}+\frac{\tilde{U}_{down0}}{\tau}=\frac{U_{max}}{\tau}-\frac{kt'}{\tau} \quad (S7)$$

$$\frac{d\tilde{U}_{down1}}{dt'}+\frac{\tilde{U}_{down1}}{\tau}=-\beta\tilde{U}_{down0}^2\frac{d\tilde{U}_{down0}}{dt'} \quad (S8)$$

Solving equations (S5) and (S7), we obtain zero approximations

$$\tilde{U}_{up0}(t)=-U_{max}+k(t-\tau)+A_{up}exp\left(-\frac{t}{\tau}\right) \quad (S9)$$

$$\tilde{U}_{down0}(t')=U_{max}-k(t'-\tau)+A_{down}exp\left(-\frac{t'}{\tau}\right), \quad (S10)$$

where $A_{up} = 2k\tau$ and $A_{down} = -2k\tau$ are constants of integration, the values of which are found from periodic initial conditions $\widetilde{U}_{up0}(t=0) = \widetilde{U}_{down0}\left(t' = \frac{T}{2}\right)$, $\widetilde{U}_{down0}(t'=0) = \widetilde{U}_{up0}\left(t = \frac{T}{2}\right)$.

Substituting (S9) and (S10) neglecting exponentials, into equations (S6) and (S8), we obtain two linear equations for the first approximation

$$\frac{d\widetilde{U}_{up1}}{dt} + \frac{\widetilde{U}_{up1}}{\tau} = -\beta k[(U_{max} + k\tau)^2 - 2kt(U_{max} + k\tau) + (kt)^2] \qquad \text{(S11)}$$

$$\frac{d\widetilde{U}_{down1}}{dt\prime} + \frac{\widetilde{U}_{down1}}{\tau} = \beta k[(U_{max} + k\tau)^2 - 2kt'(U_{max} + k\tau) + (kt')^2] \qquad \text{(S12)}$$

Substituting (S9), (S10) and particular solutions of linear equations (S11), (S12) into equations (S3) and (S4), we obtain expressions for the voltage across the capacitance C_1 of the viscoelastic branch for the upward and downward half-cycles of the command triangular voltage

$$\widetilde{U}_{up}(t) = (-U_{max} - k\tau + kt) + A_{up}exp\left(-\frac{t}{\tau}\right) - \beta k\tau\left[U_{max}{}^2 + 4U_{max}k\tau + 5(k\tau)^2\right] + 2\beta k\tau(U_{max} + 2k\tau)kt - \beta k\tau(kt)^2, \qquad \text{(S13)}$$

$$\widetilde{U}_{down}(t') = (U_{max} + k\tau - kt') + A_{down}exp\left(-\frac{t\prime}{\tau}\right) + \beta k\tau\left[U_{max}{}^2 + 4U_{max}k\tau + 5(k\tau)^2\right] - 2\beta k\tau(U_{max} + 2k\tau)kt' + \beta k\tau(kt')^2. \qquad \text{(S14)}$$

2S. Solution of the differential equation for a ferroelectric

Nonlinear charge-voltage characteristics for a membrane $q = C_0U + \frac{\beta}{3}C_0U^3$ and a ferroelectric $U = \frac{1}{C_0}q + \frac{1}{C_0}\frac{\beta}{3}q^3$ are inverse functions. Let us find the nonlinear current response of the equivalent circuit with a ferroelectric to the upward half-cycle of the triangular command voltage and compare it with the expression (13) without taking into account the exponents. Differential equation for the charge in a non-linear circuit with delayed polarization:

$$r\frac{dq}{dt} + \frac{1}{C_0}q + \frac{1}{C_0}\frac{\beta}{3}q^3 = -U_m + kt,\ 0 \le t \le \frac{T}{2} \qquad \text{(S15)}$$

we look for the solution of equation (S15) in the form of a series in powers of the small parameter:

$$q(U) = q_0 + \mu q_1 + \cdots \qquad \text{(S16)}$$

Assuming in (S15) $\frac{dq}{dt} = k\frac{dq}{dU}$; $U = -U_m + kt$; $\tau = rC_0$ we obtain two linear differential equations for describing processes in the nonlinear branch of the equivalent circuit for a ferroelectric.

$$\frac{dq_0}{dU} + \frac{q_0}{k\tau} = \frac{UC_0}{k\tau} \quad \text{(S17)}$$

$$\frac{dq_1}{dU} + \frac{q_1}{k\tau} = -\frac{1}{k\tau}\frac{\beta}{3}{q_0}^3 \quad \text{(S18)}$$

Substituting partial solutions of equations (S17) and (S18) into (S16), we obtain an expression for the charge

$$q = C_0(U - k\tau) - \frac{\beta}{3}{C_0}^3U^3 + 2\beta C_0^3U^2k\tau - 5\beta C_0^3U(k\tau)^2 + \frac{16}{3}\beta C_0^3(k\tau)^3 \quad \text{(S19)}$$

The current response to the upward half-cycle of the triangular voltage without taking into account transients:

$$I_{0up} = k\frac{dq}{dU} + gU == kC_0\left(1 - \beta_{exp}C_0^2(U^2 + 5(k\tau)^2)\right) + \left(g + 4\beta_{exp}kC_0^3k\tau\right)U \quad \text{(S20)}$$
